# Densitometry of STR-PAGE for donor chimerism in acute leukemia’s: A simple method for routine use

**DOI:** 10.12669/pjms.40.5.9216

**Published:** 2024

**Authors:** Ayesha Nayyar, Suhaib Ahmed

**Affiliations:** 1 Ayesha Nayyar, M. Phil, PhD Department of Pathology, Islamic International Medical College, Riphah International University, Islamabad, Pakistan; 2 Suhaib Ahmed, FCPS, PhD Department of Pathology, Islamic International Medical College, Riphah International University, Islamabad, Pakistan

**Keywords:** DC, STR, PCR, PAGE, Densitometry, Informative allele

## Abstract

**Objective:**

To evaluate a PCR based method of polyacrylamide gel electrophoresis of short tandem repeats and its quantification for detecting donor chimerism after haematopoietic stem cell transplantation in acute leukaemias.

**Methods:**

The descriptive study was conducted at Genetic Resource Centre (GRC) Lab Rawalpindi from Feb 2018 - Nov 2020. A total of twenty patients with acute leukaemias having undergone HSCT were selected and assessed for the analysis of chimerism status. DNA extraction from the whole blood was done by chelex method and short tandem repeats were amplified by using conventional STR- PCR assay. Electrophoresis was carried out and 6% polyacrylamide gels were used for the resultant amplified DNA products and then followed by their densitometry. These patients had undergone HSCT from Pakistan Institute of Medical Science and Armed Forces Bone Marrow Transplant Centre.

**Results:**

The peaks in the PAGE densitometry represented the donor chimerism in all post transplant samples of the patients.

**Conclusion:**

Our study showed that densitometry of STR PCR PAGE is a useful and cheaper method for demonstration of donor chimerism in acute leukaemia patients having undergone HSCT. Hence this method can be a valuable option in the monitoring of chimerism status in these patients and therefore helps in preventing graft failure by fast and early treatment strategies for these patients.

## INTRODUCTION

Short tandem repeats (STRs), also known as Microsatellites are small sequences of DNA (two to six base pairs) which are tandemly repeated and these repeat units are different among individuals, making them highly distinguishable^1^ and are highly useful and widely used in human identity testing applications.^2^,^3^

Analysis of short tandem repeats (STR-PCR) is thought to be the standard PCR based assay for detection of chimerism^4^ Chimerism refers to the presence of mixture of DNA of both the donor and recipient after a hematopoietic stem cell transplant as HSCT has been widely used for the cure of patients suffering from malignant and nonmalignant hematological disorders and considered.^5^ Nevertheless testing of hematopoietic chimerism influences clinical decision and therapeutic intervention in patients after haematopoietic stem cell transplantation but HSCT may get failed due to relapse, and graft-versus-host disease (GVHD).^6^ Estimation of chimerism pattern has a central part in monitoring of patients having undergone stem cell transplant^7^ and short tandem repeats (STR-PCR) are considered the standard PCR based method for detecting chimerism.^8^ The method of STR-PCR using a genetic analyzer is sensitive but costly.

The analysis of polyacrylamide gel electrophoresis with subsequent densitometric quantification of the DNA fragments is another alternative which is cheaper than the use of genetic analyzer.^9^ PCR of short tandem repeats using PAGE by genetic analyzer is having lesser sensitivity with high coefficient of variation.^10^ The troubles most likely faced using genetic analyzer are the artifacts of polymerase slippages i.e stutter peaks, ultimately interfering with the analysis of chimerism pattern.^11^

Hence by analyzing the samples of peripheral blood or bone marrow of the recipient over time may help in detecting the patterns of Chimerism by conventional STR-PCR PAGE after a stem cell transplant in several malignant and non-malignant hematological diseases. Therefore a continuous monitoring of chimerism is of primary importance in the effective tailoring of the treatment strategies wherever required.^12^ This paper provides a simple, reliable and successful use of STR-PCR by densitometric evaluation of donor chimerism pattern in acute leukaemias.

## METHODS

This descriptive study was conducted at Genetic Resource Centre Rawalpindi. The Twenty post-transplant samples of the patients with acute leukaemias who had their stem cell transplanr were studied. About 3-ml of peripheral blood was taken in EDTA container. DNA was extracted using Chelex™ method.^11^ To flank the repeat units in the gene regions, specific STR primers were designed and PCR conditions for STR were same as described by Ahmed.^11^ Each sibling pair (donor and recipient) were first run at ten different autosomal STR loci (Table-I). The informative locus was identified i.e. donor and recipient had at least one exclusive allele. The donor and the recipient post-transplant samples were run for the informative STR locus. The STR amplified products were analysed on 10 X 20 cm polyacrylamide gels (PAGE) after silver staining. The results of PAGE results were photographed and were analysed by a software image analysis (http://thal-it.com). The donor Chimerism was then calculated and demonstrated as percent of complete donor Chimerism.

**Table I:** Relative informative-ness of eight STR loci in the 20 sibling (donor/recipient) pairs.

STR	Loci Informative in sibling pairs	%
***Common***		
D5S818	8/20	40.0 (0.40)
D3S1358	8/20	40.0 (0.40)
D7S820	5/20	25.0 (0.25)
D8S1179	5/20	25.0 (0.25)
***Uncommon***		
FGA	4/20	20.0 (0.20)
TH01	3/20	15.0 (0.15)
TPOX	2/20	10.0 (0.10)
D13S317	2/20	10.0 (0.10)
***Rare***		
D18S51-R	1/20	5 (0.05)
D21S1411-F	1/20	5 (0.05)

### Ethical Approval:

The study was approved by the institutional ethical review board, Faculty of Medical Sciences, Riphah International University Islamabad-Pakistan (Ref. # Riphah/IIMC/IRC/23/3036).

## RESULTS

### Informative STR loci in the Donor/Recipient pairs (sibling pairs):

All the twenty sibling pairs when analyzed by STR-PCR showed that at least one informative STR locus was found in every pair (Table-I). The informative STR loci were divided into “Common”, “Uncommon” loci and “rare”. In each donor/recipient pair the number of informative loci varied from 1 to 8. The usefulness of each STR locus, defined by the number of informative loci, varied from 5% to 40% as shown in Table-I.

### Donor Chimerism by STR PCR:

**Fig.1:**
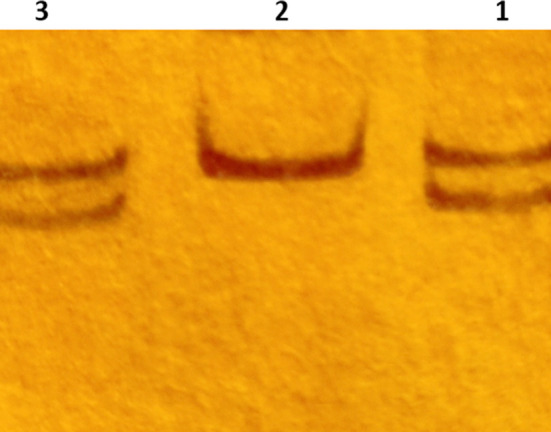
Silver stained polyacrylamide gel electrophoresis of STR PCR at D5S818 locus. Lane 1 shows two distinct alleles in the recipient pre-transplant sample. Lane 2 shows two alleles of the same size (homozygous) appearing as a single thick band in the donor sample. Lane 3 shows the recipient post-transplant sample with two alleles but the intensity of the shorter allele (lower band) is significantly less than the larger allele (upper band). The reappearance of the recipient specific allele (lower band) indicates a reappearance of the recipient’s tissue (decreasing chimerism).

The results achieved from STR-PCR in all the sibling pairs were obtained and read from the silver stained polyacrylamide gels. Densitometric evaluation of the polyacrylamide gel of STR-PCR was done later on. (Fig.1 to Fig.2.2). The densitometric evaluation of PAGE clearly showed donor chimerism in post transplant samples of the patients in regards to their percentages estimated. The overall results of the 20 sibling pairs and the level of chimerism in these pairs ranged from 13.2% to 98.9%.

**Fig.1.1:**
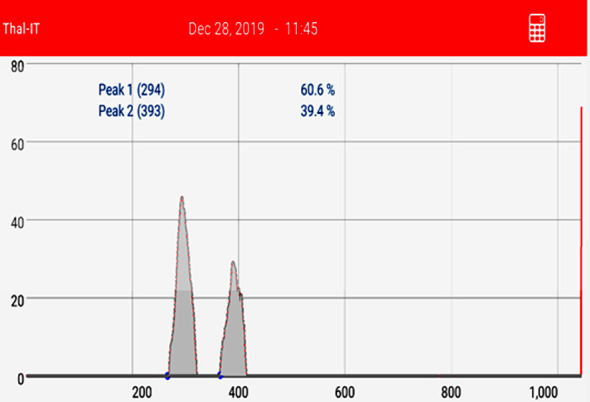
Densitometric evaluation of the polyacrylamide gel electrophoresis of the recipient’s post-transplant sample shown in the figure 1. Peak 2 (39.4%) is the recipient specific allele and its reappearance indicates a reappearance of the recipient’s tissue (decreasing chimerism). The donor chimerism in this case was reported as 60.6% (60.6% donor component and 39.4% recipient component).

**Fig.2:**
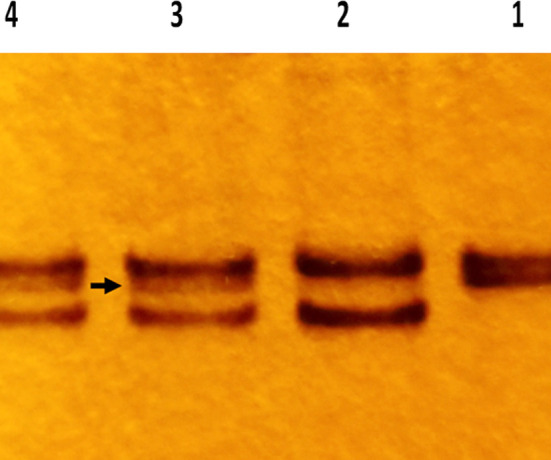
Silver stained polyacrylamide gel electrophoresis of STR PCR at D3S1358 locus. Lane 1 shows two closely packed alleles in the recipient pre-transplant sample. Lane 2 shows two distinct alleles in the donor sample. Lanes 3 & 4 show the recipient post-transplant samples with three alleles. The middle allele (indicated by arrow) is the recipient’s exclusive allele. Its reappearance in the post-transplant sample indicates a reappearance of the recipient’s tissue (decreasing chimerism).

**Fig.2.2:**
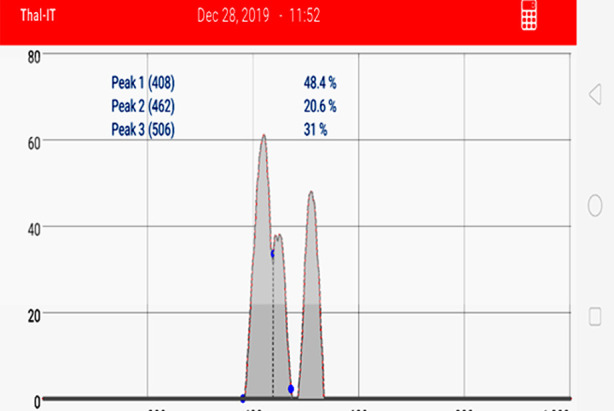
Densitometric evaluation of polyacrylamide gel electrophoresis of the recipient’s post-transplant sample shown in the figure 2. Peak 2 (20.6%) is the recipient specific allele and its reappearance in a post-transplant sample indicates a reappearance of the recipient’s tissue (decreasing chimerism). The donor chimerism in this case was reported as 79.4% (79.4% donor component and 20.6% recipient component).

### Donor Chimerism by STR-PCR:

Results of donor chimerism by STR PCR in the 20 sibling pairs were read from the silver-stained polyacrylamide gels and their densitometry.

## DISCUSSION

 Currently Haematopoietic stem cell transplant is considered to be an effective and efficient therapeutic strategy for managing different hematologic malignancies, but alongwith is compounded by various complications, such as disease recurrence, toxicity related to treatment related toxicity, Graft rejection and GVHD.^13^ The main cause of failure of haematopoietic stem cell transplant is relapse inspite of continuous improvement in this therapeutic modality especially in patients with acute leukemia. As recommended by EuroChimerism consortium PCR analysis of STR is the standard method for estimation of quantitative chimerism in the prediction of relapses.^14^

Regarding routine chimerism monitoring there are promising investigations on the new methods; however, for monitoring of chimerism status currently STR-PCR is the established method which not only determines the type of chimeras, but also determines the percentage of both donor and recipient cells.^8^ Hence after HSCT reappearance or persistence of recipient cells can indicate the recurrence of the recipient’s hematopoietic cells or presence of malignant cells or both.^15^ Many researchers have proved that the PCR analysis of short tandem repeat profiles of DNA sequences and single nucleotide polymorphisms analysis with real-time quantitative PCR based methods are major techniques used for detection of chimerism.^12^,^16^ The STRs have an advantage over other molecular markers for estimating the chimerism because of increase number of allels and low amount of DNA required in a PCR reaction.^17^ The detection limit of STR-PCR for recipient chimerism is ranging from 1 to 10% in few studies. Although technical variability among laboratories may be noticeable.^18^,^19^

Few researchers found out that by using STR markers in 56 children with ALL after HSCT early detection of chimerism can be found out easily.^20^ Hence for analysis of chimerism, the preferred technique should be easy to perform, rapid, cheap, easily interpretable and applicable to every patient. In addition, results should have less variation between the samples and should be highly reproducible. However these consideration should be taken into account for analyzing the exact quantification of donor/recipient DNA for assessing engraftment of the donor DNA and diagnosing the failure of graft in an early manner.^4^

In a study by Andrikovics et al a total of 12 STR markers are needed to obtain 95% informativity for the analysis of chimerism.^21^ A group of researchers in their study of 230 patients of malignant hematological disorders found out that five STR informative markers were able to find chimerism in all patients except ten patients who deceased and also suggested that STR is more informative in detecting mixed chimerism as compared to other method.^22^ A Japanese study suggested that using 20 STR markers and the KMR kit markers the informativity in donor/recipient pairs for estimation of chimerism may be detected in a large variety of donor/recipient pairs.^23^

Few researchers have suggested that at least 40 markers are required to discriminate a large number of the donor recipient pairs. 24 A group of researchers in their study have observed the status of chimerism by using STR markers in 39 donor/recipient pairs and found chimerism in all the pairs.^25^

## CONCLUSION

Our study demonstrates that STR -PCR using densitometric evaluation is a simple and favourable technique without use of any special instrumentation and expensive reagents making it suitable for a use in routine laboratory practices. Hopefully this will help in analysis of the outcomes after hematopoietic stem cell transplantation in patients suffering from acute leukaemias.

### Authors Contribution:

**AN:** literature search, study design and concept, data collection, data analysis, data interpretation, drafting.

**SA:** Study design and concept, data analysis, data interpretation, Critical Review, Final approval.
